# Everybody's Page

**Published:** 1888-05-05

**Authors:** 


### Everybody's Page.

For use in bed-rooms and libraries colza oil is the best, because, as it contains oxygen, it impoverishes the air less, and its waste products are also less destructive than those of gas, but it is about three times as costly.

Green tea is sometimes "faced," as it is called—that is, it is powdered on the surface with different ingredients for the purpose of communicating to the leaves the admired green appearance. Prussian blue, indigo, plaster of Paris, slacked lime, graphite, and French chalk, have all been employed for this purpose.

At the close of the convivial meetings which doctors now and again indulge in, the toast "Floreat res medica" is always proposed and drunk with proper enthusiasm. This, of course, literally means "may medicine flourish," but by a late physician of Edinburgh it was perhaps as aptly translated "long life and ill-health."

Not long ago portions of a dry parchment-like substance were found nailed to the doors of certain very old churches. On these being removed minute hairs were observed springing from the surface, which on examination proved to be of human origin. The parchment was thus proved to be human skin, and represented the hides of some unfortunate individuals who had been flayed for sacrilege.

There is reason to believe that fair-haired women are at a slight discount in the matrimonial market. Statistics would seem to show that brunettes are more successful in obtaining husbands in the proportion of about three to two. Poets have a proverbial preference for fair hair; no poem is complete without a flaxen-haired maid; but prosaic mortals in search of a wife seem, upon the whole, to prefer the brown and the black. According to Dr. Beddoe, the result of this "conjugal selection" appears to be that in some parts of England fair hair is slowly disappearing, whilst dark hair is becoming more prevalent.

Milk is frequently contaminated on its way to the consumer. Particles and germs are floating in the air which easily get into a badly-covered can, and are thus a source of danger. Cases are recorded where disease has arisen from this cause, and where the strictest and most minute investigation has proved that this was the only source of contamination. To obviate such a contingency Dr. McVail of Glasgow has introduced his patent vented can, where, by special precautions, the can and milk are freed from germs, and the air which enters the vessel to supply the place of the milk taken out by a tap at the bottom passes through a porous substance, which thus filters it. If there is any reason to suspect the purity of milk, it should in all cases be boiled in order to kill the germs.

A new development of the automatic principle which has been so successfully applied to the machines which supply the public with post-cards, chocolate and cigars at railway stations, has been applied in a northern town. It is well known that the poor do not often burn gas, not because they have any æsthetic preference for lamps, but because they feel the burden of a quarterly payment more than they do the expenditure of a few pence weekly for oil. The new machine is an automatic gasmeter. One of these is placed in a house, and when the occupant wishes light he drops a penny into a slot. By an ingenious arrangment the weight of the penny forces a certain number of cubic feet of gas—just a pennyworth—into the pipes, which the tenant can use at will. Once a week the collector of the gas company calls, and takes the pennies out of the locked receptacle into which they drop.

Avoid houses in which artificial light is required during the day. Choose, if possible, a street with a good exposure to the sun. Follow the advice of the old proverb, "Live on the sunny side of the street, where the doctor never comes." Do not take a house which never receives the direct rays of the sun.

According to Professor Bischoff, of Munich, the difference between the average brain-weight of men and women is 10½ per cent. Much of this is undoubtedly due to difference in stature, a tall person having, *cæteris paribus*, a larger brain than one less in height; partly, however, it is attributable to inferior mental training.

Any system of ventilating an apartment is most materially aided by a fire in the room, and the great safety in the occupancy of many of our small houses lies in the fact that the living and sleeping rooms are one and the same, and that the fire required for daily wants does triple service in the cooking of food, the warming of the apartment, and the ventilation of the home.

The difference, according to Le Bon, between the average capacity of the skulls of male and female Parisians is almost double that found to obtain between the skulls of the male and female inhabitants of Ancient Egypt. Civilisation, by giving increased exercise, especially to the male brain, has there is good reason to believe, gradually produced that increase of brain capacity which now distinguishes the civilized from the savage races of mankind.

Dr. Hayes, the celebrated American Arctic explorer, declared that alcohol taken for the purpose of resisting cold was "not only completely useless, but positively injurious"; while the experience of the officers who accompanied the Red River expedition under Sir Garnet Wolseley in 1870 was, that while the troops had as arduous work to perform as ever fell to the lot of British soldiers, they continued throughout in excellent health, although not a drop of drink was given, the only beverage being tea.

The first Bill for taking a census was introduced into Parliament in 1753, but defeated by such language as the following: Mr. Thornton, member for the city of York, said —"I did not believe that there was any set of men, or, indeed, any individual of the human species, so presumptuous and so abandoned as to make the proposal we have just heard." And Mr. Matthew Ridley, member for Newcastle-on-Tyne, said—"The people looked upon the proposal as ominous, and feared lest some public misfortune or an epidemical distemper should follow the numbering."

The Orphan House at Lahr, in the Duchy of Baden—which has been built upon, endowed with, and is sustained by, the ends of cigars—has been frequently described. It originated with the editor of that most popular of German year-books, the *Lahrer Kalender*, and it has its busy collectors and contributors of cigar ends in every German town and among the German communities on the other side of the Atlantic. A new society of benevolent collectors, according to the *Kleine Zeitung*, is now at work. The members gather together all the old used corks they can find—it matters not how damaged they are—and forward them to the central depot, from whence they are sent to linoleum factories, as the collected cigar ends are sent to tobacco factories. The yield from the corks, it is said, promises to be much more profitable than that from the cigar ends, and some poor folk have been employed (by a middleman?) in collecting them.

